# Association of fibroblast growth factor 10 with the fibrotic and inflammatory pathogenesis of Graves’ orbitopathy

**DOI:** 10.1371/journal.pone.0255344

**Published:** 2021-08-12

**Authors:** Sun Young Jang, Soo Hyun Choi, Don Kikkawa, Eun Jig Lee, Jin Sook Yoon

**Affiliations:** 1 Department of Ophthalmology, Soonchunhyang University Bucheon Hospital, Soonchunhyang University College of Medicine, Bucheon, Republic of Korea; 2 Department of Ophthalmology, Severance Hospital, The Institute of Vision Research, Yonsei University College of Medicine, Seoul, Republic of Korea; 3 Division of Oculofacial Plastic and Reconstructive Surgery, University of California San Diego, La Jolla, California, United States of America; 4 Department of Endocrinology, Severance Hospital, Institute of Vision Research, Yonsei University College of Medicine, Seoul, Republic of Korea; University of North Texas Health Science Center, UNITED STATES

## Abstract

**Purpose:**

The role of fibroblast growth factor (FGF) in orbital fibroblasts (OFs) is rarely known. In this study, we investigated the effect of FGF10 on fibrosis and the inflammation mechanism of Graves′ orbitopathy (GO).

**Methods:**

Orbital tissue from GO (n = 15) and non-GO (n = 15) was obtained for this study. The mRNA and protein expression levels of FGF10 and FGF receptor 2b (FGFR2b) in orbital tissue were determined by real-time polymerase chain reaction, western blot analysis, and confocal microscopy. The effects of FGF10 on transforming growth factor (TGF)-β1 induced fibrotic proteins and interleukin (IL)-1β- or tumor necrosis factor (TNF)-α- induced inflammatory proteins were investigated using recombinant human (rh) FGF10 and small interfering (si) RNA transfection against FGF10.

**Results:**

FGF10 and FGFR2b mRNA expression levels were significantly lower in GO orbital tissues than in non-GO orbital tissues (p = 0.009 and 0.005, respectively). Immunostaining of FGF10 in orbital adipose tissues showed differences in FGF10 expression between GO and control samples. Immunostaining of FGF10 was very weak in the orbital tissues of GO patients. TGF-β1-induced fibronectin, collagen Iα, α-smooth muscle actin protein expression in GO OFs was attenuated by rhFGF10 treatment and increased by knockdown of FGF10 via siFGF10 transfection. Similarly, IL-1β- or TNF-α-induced IL-6, IL-8, and cyclooxygenase-2 protein production in GO OFs was either blocked by rhFGF10 treatment or further upregulated by inhibition of FGF10 via siFGF10 transfection.

**Conclusions:**

Our data demonstrate that FGF10 has beneficial effects on the inflammatory and fibrotic mechanisms of GO in primary cultured OFs, providing new insights into GO pathology and the discovery of FGF10 as a promising novel therapeutic application for the treatment of GO.

## Introduction

Graves′ orbitopathy (GO) is an inflammatory autoimmune disease of the extraocular muscles and orbital fat or connective tissue, generally observed in patients with Graves’ disease [1]. Although the pathogenesis of GO remains unclear, orbital fibroblasts (OFs) play a key role in the disease process. OFs express the thyroid-stimulating hormone receptor (TSHR) and activation of the OFs by inflammatory mediators and stimulatory autoantibodies directed against the TSHR and insulin-like growth factor-1 receptor enhance inflammation, hyaluronic acid production, adipogenesis, and fibrosis in GO patients [2, 3]. Fibrosis can be followed by inflammation as a part of the wound healing process [4, 5]. Although the clinical course of GO is variable, usually an initial active inflammatory phase, lasting 6 to 24 months, is followed by a chronic fibrosis phase [6].

Fibroblast growth factor (FGF) was discovered as a mitogen for cultured fibroblasts [7]. FGFs play important roles during embryonic development and adult organisms by regulating cell proliferation, differentiation, migration, survival, angiogenesis, and organogenesis [8, 9]. Over the last few years, 22 distinct FGFs have been identified. Although the roles of FGFs in OFs are rarely known, basic FGF (FGF2) has been proposed to contribute to GO [2, 10, 11]. Previous reports demonstrated that serum and tissue FGF2 levels were increased in patients with active GO, suggesting that FGF2 could reflect the ocular inflammatory activity in GO patients. However, besides FGF2, the role of FGFs in OFs has rarely been studied. Based on phylogenetic analysis, FGF family is further divided into seven subfamilies: FGF1 and FGF2 (FGF1 subfamily); FGF4, FGF5 and FGF6 (FGF4 subfamily); FGF3, FGF7, FGF10 and FGF22 (FGF7 subfamily); FGF11, FGF12, FGF13 and FGF14 (FGF11 subfamily); FGF19, FGF21, and FGF23 (FGF19 subfamily) [12]. FGF7, also known as a keratinocyte growth factor-1 (KGF1) and FGF10 (KGF2), interacts with one of the FGF receptors (FGFRs), expressed particularly by epithelial cells, and may serve as a protective factor for epithelial tissue.

FGF10, belonging to the FGF7 subfamily, activates key intracellular signaling pathways in several cell types, leading to the modulation of cell proliferation, differentiation, and migration during wound healing and tissue repair [13]. Gupte et al. [14] reported that overexpression of FGF10 during both inflammatory and fibrotic phases attenuates bleomycin-induced pulmonary fibrosis in mice. Sun et al. [15] showed that reduced expression of FGF10/FGFR2 was found in rats with di-n-butyl phthalate-induced renal fibrosis. FGF10 has been detected in the cytoplasm of cultured prostate stromal cells [16] and the nucleus of urothelial cells [17], suggesting that different subcellular localization of FGF10 can be possible underlie the specificity of FGF10 signaling in different cell type. In epithelial cells, upon FGF10 binding, FGFRs dimerize, and several intracellular tyrosine residues are trans-autophosphorylated. FGFR engages multiple and complex signaling pathways via adaptor proteins in epithelial cells [18].

As GO is an inflammatory and fibrotic disease, it was hypothesized that FGF10 might participate in its pathogenesis. However, to the best of our knowledge, few previous reports regarding the association between FGF and GO mechanisms. Of the various types of FGFs, we focused on FGF10 and aimed to determine whether FGF10 decreased the expression of molecules associated with inflammation and fibrosis in an in vitro model of GO.

## Materials and methods

### Cultures

Orbital adipose tissues were obtained from GO patients as surgical waste during orbital decompression for exophthalmos (n = 15; 6 men, 9 women; aged 25–65 years). The decompression surgery was performed in an inactive phase of GO for rehabilitative purposes, and thyroid hormones were well controlled during surgery. Clinical activity scores at the time of surgery were less than 3. None of the patients with GO had received steroid or radiotherapy treatment for at least 6 months prior to the surgery. Non-GO orbital tissue samples were collected during eyelid or orbital surgery from age- and sex-matched non-GO subjects with no history or clinical evidence of thyroid disease (n = 15; 6 men, 9 women; aged 23–71 years). Three patients underwent orbital surgery for other non-inflammatory diseases and 12 patients underwent cosmetic upper and lower blepharoplasty. The study was approved by the Institutional Review Board of Severance Hospital, Yonsei University College of Medicine. Written informed consent was obtained from all patients who were enrolled in this study. This study followed the tenets of the 1975 Declaration of Helsinki.

The established methods of the primary culture were used for the primary culture of OFs [5, 19]. Briefly, the adipose tissue was minced and placed in 1:1 Dulbecco’s modified Eagle’s medium (DMEM): F12 medium with 20% fetal bovine serum (FBS) and penicillin/gentamycin. Fibroblasts were cultured from the explants, and then monolayers were passaged serially with trypsin/ethylenediaminetetraacetic acid (EDTA). Cultures were maintained in DMEM: F12 with 10% FBS and antibiotics. Fibroblasts between the second and fifth passages were used for analysis.

### Reagents

DMEM: F12 and penicillin-streptomycin were purchased from WelGENE Inc. (Daegu, Korea). FBS was purchased from Invitrogen (Carlsbad, CA, USA). FGF10 antibody, recombinant human (rh) FGF10, interleukin (IL)-1β, tumor necrosis factor (TNF)-α, and transforming growth factor (TGF)-β1 were purchased from R&D Systems (Minneapolis, MN, USA). Small interfering (si) FGF10 was purchased from Horizon Discovery Ltd. (Lafayette, CO, USA). Negative controls were purchased from Invitrogen. We purchased fibronectin from BD (Franklin Lakes, NJ, USA) and α-smooth muscle actin (SMA) antibody was purchased from Sigma-Aldrich (St. Louis, MO, USA). IL-6 antibody was purchased from Novus Biologicals (Centenial, CO, USA). Collagen Iα (COL1A) and IL-8 antibodies were purchased from Abcam (Cambridge, UK). Matrix metalloproteinase (MMP)-2, cyclooxygenase (COX)-2, extracellular signal-regulated kinase (ERK), and phospho-ERK were purchased from Cell Signaling Technology (Danvers, MA, USA). Collagen IIIa (COL3A) and β-actin were purchased from Santa Cruz Biotechnology (Logan, UT, USA).

### Quantitative real-time polymerase chain reaction (qRT-PCR)

Orbital tissue specimens (n = 10 for GO, n = 10 for non-GO) were used to compare the mRNA levels of FGF10 and FGFR2 using qRT-PCR. In primary cultured OFs from GO and non-GO cells, mRNA expression levels of *fibronectin*, *COL1A*, *and α-SMA* were measured using qRT-PCR. Orbital tissues were homogenized with a tissue homogenizer (Precellys^®^ 24; Bertin Instruments, Montigny-le-Bretonneux, France) using a Precellys lysing kit (Bertin Instruments) with Trizol (Invitrogen, Thermo Fisher Scientific, Waltham, MA). RNA was extracted from the cells using TRIzol (Invitrogen), and the RNA concentration was determined using NanoDrop (Thermo Fisher Scientific). A total of 1 μg of mRNA was reverse-transcribed into cDNA (SensiFAST cDNA Synthesis Kit; Meridian Life Science, Memphis, TN, USA) and amplified with SYBR green PCR master mix (TaKaRa Bio, Shiga, Japan) and Taq Man^™^ Gene Expression Master Mix (Thermo Fisher Scientific) using the LightCycler^®^ 480 System (Roche, Basel, Switzerland). All PCR reactions were performed in triplicates, and all samples were normalized to glyceraldehyde-3-phosphate dehydrogenase (GAPDH) expression levels. The primers used for amplification were as follows: *FGF10*, 5′-GAA GGA GAA CTG CCC GTA CA-3′ (forward) and 5′-GGC AAC TCC GAT TTC TAC T-3′ (reverse); *FGFR2b* 5′-GCT GGC TCT GTT CAA GAC-3′ (forward) and 5′-GGC CTG CCC TAT ATA ATT GGA-3′ (reverse); *GAPDH* 5′- TGC TGT AGC CAA ATT CGT TG-3′ (forward) and 5′- CAC CCA CTC CAC CTT T-3′ (reverse). TaqMan gene expression assay targets: Hs00426835_g1 for *α-SMA*, Hs01549976_m1 for *fibronectin*, Hs00164004_m1 for *COL1A*, and Hs99999905_m1 for *GAPDH* (Thermo Fisher Scientific).

### Western blots

Western blot analyses of FGF10, fibronectin, COL3A, COL1A, α-SMA, MMP-2, IL-6, IL-8, cyclooxygenase (COX)-2, intercellular adhesion molecule (ICAM)-1, total ERK and phospho-ERK were performed as described previously [19, 20]. The relative densities were quantified and normalized to that of β-actin in the same sample. Full length gels representative of Western blot analysis were provided in S1–S4 Figs.

### ELISA analysis

FGF10 expression was analyzed using a human cytokine ELISA kit (Abbexa Ltd., Cambridge, UK) according to the manufacturer’s protocol. Levels of FGF10 in GO OFs under IL-1β, TGF-β, and TNF-α stimulation were determined. ELISA was used to determine levels of secreted FGF10 in OF culture differentiation medium. After incubation for various times (24, 48, 72 h) with IL-1β (10 ng/ml), TGF-β (5 ng/ml) or TNF-α (10 ng/ml), the culture supernatants were collected and ELISA was used to determine FGF10 levels.

### rhFGF10 and siRNA transfection against FGF10

Cells were treated with rhFGF10 (100 ng/ml) or transfected with siFGF10 (100 ng/mL, 24 h) following the manufacturer’s instructions. Approximately 70% of confluent OFs from patients with GO and non-GO were prepared in 6-well plates. Negative control siRNA or FGF10 siRNA was transfected with a commercial reagent (TransIT-siQUEST^®^ Reagent; Mirus Bio, Madison, WI, USA). After pretreatment of FGF10 or transfection with siFGF10, cells were incubated with or without 10 ng/mL of IL-1β, TNF-α, or 5 ng/mL of TGF-β for 16 h.

### Immunofluorescence (IF) staining

For IF staining, sections of paraffin-embedded orbital tissue were deparaffinized and rehydrated. After blocking with 3% BSA, the sections were incubated with anti-FGF10 antibody (Santa Cruz Biotechnology) and Alexa Fluor Fluor-488 anti-mouse IgG (Molecular Probes^™^, Thermo Fisher Scientific). For nuclear counterstaining, Fluoroshield^™^ with 4’,6-diamidino-2-phenylindole dihydrochloride (DAPI; Sigma-Aldrich) was used. Fluorescence images were captured with a microscope (IX73; Olympus Optical Co., Ltd., Tokyo, Japan) with a charge-coupled device camera (DP71; Olympus Optical Co., Ltd.).

### Statistics

All experiments were performed at least three times with samples from each individual, and sample assays were performed in duplicates. All results are expressed as means ± standard deviation. Independent t-tests and one-way analysis of variance (ANOVA) with post-hoc analysis were used in this study. A statistical program (SPSS for Windows, version 26; SPSS, Chicago, IL) was used, and values of P < 0.05 were considered significant.

## Results

### GO tissues and OFs show decreased expression of FGF10

**Fig 1:**
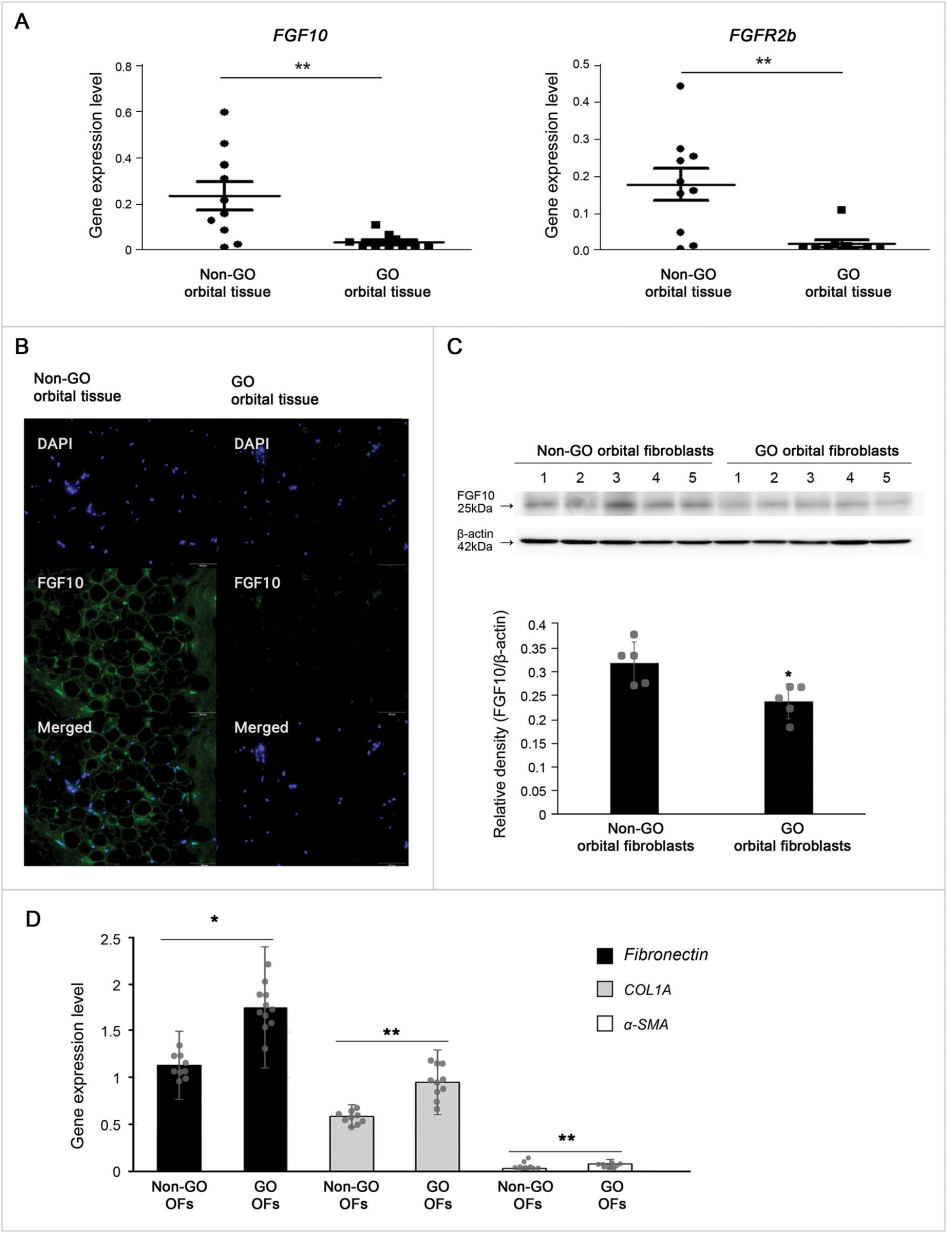
Expression of fibroblast growth factor 10 (FGF10) in Graves′ orbitopathy (GO) and in non-GO tissues and cells. (A) Expression of fibroblast growth factor 10 (FGF10) and FGF receptor 2b (FGFR2b) mRNA in GO and non-GO orbital tissues. (B) Microscopy images showing the expression of FGF10 (green) and live-cell nuclei (4’,6-diamidino-2-phenylindole dihydrochloride; blue) in GO and non-GO orbital tissues. (C) Expression of FGF10 protein in GO and non-GO orbital fibroblasts (OFs). Bar graphs show the relative density of FGF10 normalized to the level of β-actin and are represented as means ± standard deviation. (D) Gene expression of fibronectin, collagen Iα, and α- smooth muscle actin in GO and non-GO OFs. Experiments were performed at least three times using different strains. *p < 0.05, **p < 0.01.

We compared mRNA level of FGF10 and FGFR2b in GO (n = 10) and non-GO (n = 10) orbital tissues explants using RT-PCR. mRNA transcript levels of FGF10 were greater in non-GO tissue than in GO tissue (p = 0.01, independent *t*-test) as shown in Fig 1A. RNA transcript levels of FGFR2b were also greater in non-GO tissue than in the GO tissue (p = 0.005, independent *t*-test) (Fig 1A).

Microscopy images showing FGF10 (green) and live-cell nuclei (DAPI; blue) expression in GO and non-GO orbital tissue confirmed immunostaining of FGF10 in control orbital adipose tissue, which was, on the other hand, very weak in GO tissue (Fig 1B).

FGF10 proteins in primary cultured OFs were more expressed in non-GO OFs (n = 5) than in GO OFs (n = 5) in western blot analyses (Fig 1C). Densitometry results showed a statistically significant lower expressions of FGF10 in GO OFs than in non-GO OFs (p = 0.015, independent *t*-test).

To identify the unique phenotype of GO OFs, *fibronectin*, *COL1A*, and *α-SMA* gene expression was compared between GO OFs (n = 3) and non-GO OFs (n = 3) using RT-PCR. The mRNA transcript levels of *fibronectin*, *COL1A*, and *α-SMA* expression were significantly higher in GO OFs than in non-GO OFs (Fig 1D).

### Effects of TGF-β1 and rhFGF10 on fibronectin, collagen Iα, and α-SMA protein expression in GO OFs

**Fig 2:**
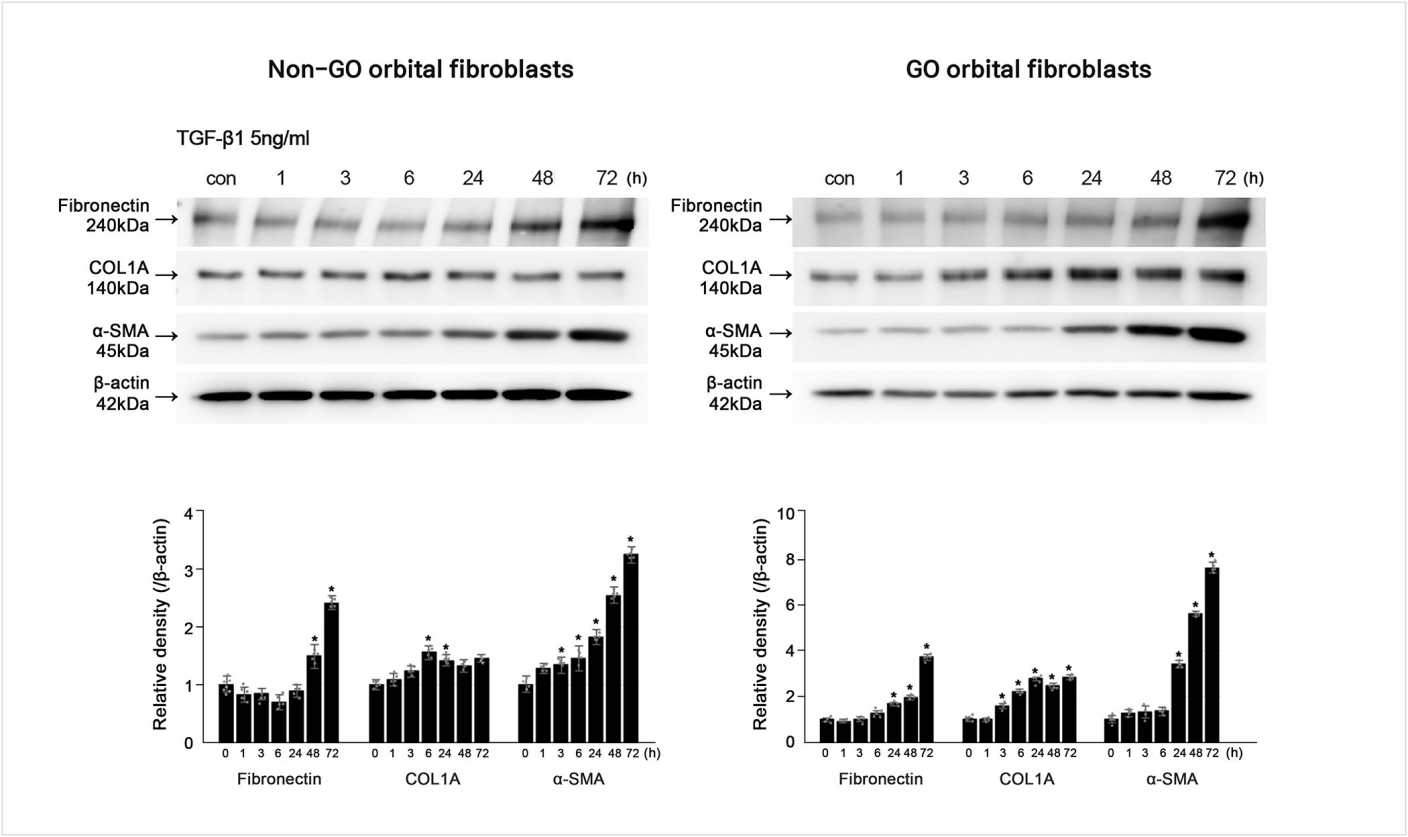
Effect of transforming growth factor (TGF)-β1 on fibrosis-related protein production in the Graves′ orbitopathy (GO) and non-GO orbital fibroblasts (OFs). After treatment of the OFs with 5 ng/mL TGF-β1 for 0, 1, 3, 6, 24, 48, and 72 h, the protein expression levels of fibronectin, collagen Iα, α-smooth muscle actin increased in a time-dependent manner. Bar graphs show the relative density of each protein normalized to the level of β-actin and are represented as mean ± standard deviation. Experiments were performed using at least cells using different strains. *p < 0.05.

Since TGF-β is known to be important for the induction of tissue remodeling and fibrosis, we investigated whether TGF-β could induce fibrotic processes in GO and non-GO OFs. By western blot analysis, we confirmed that fibrosis-related proteins, including fibronectin, collagen Iα, and α-SMA, significantly increased in GO and non-GO OFs after stimulation with TGF-β in a time-dependent manner (Fig 2).

**Fig 3:**
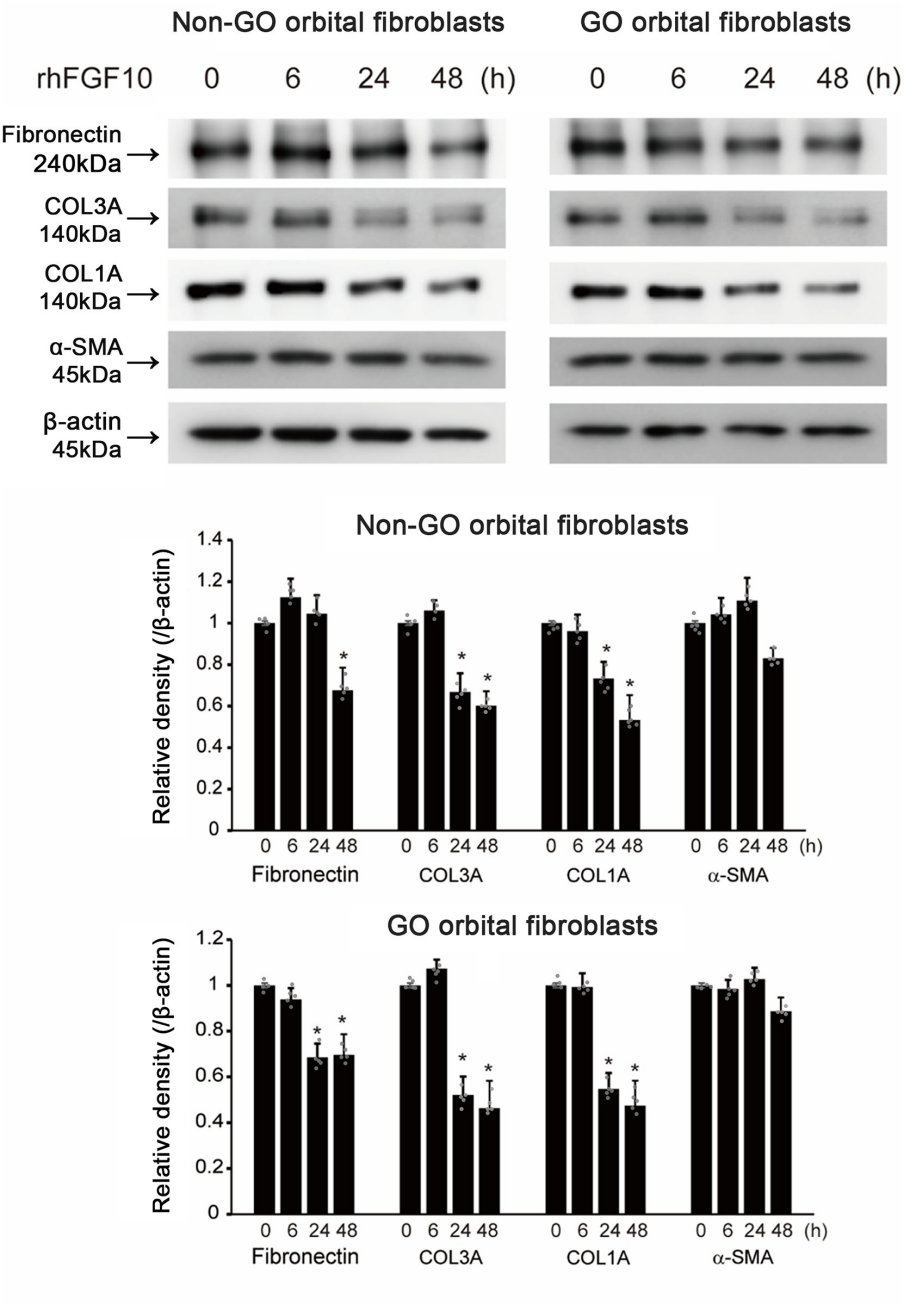
Inhibitory effect of FGF10 on Graves′ orbitopathy (GO) and non-GO orbital fibroblasts. After treatment with 100 ng/mL of rhFGF10 for 0, 6, 24, and 48 h, fibronectin, collagen IIIa (COL3A) and collagen Iα (COL1A) protein expression was significantly decreased at 24 h or 48 h. Bar graphs show the relative density of each protein normalized to the level of β-actin and are represented as means ± standard deviation. Experiments were performed using at least three cells from different strains. *p < 0.05.

To clarify the role of FGF10 in the fibrotic response, we treated GO and non-GO OFs with rhFGF-10 for different times. Treatment of GO and non-GO OFs with 100 ng/mL rhFGF10 for 24 h or 48 h significantly repressed fibronectin, COL3A, and COL1A protein expression (Fig 3).

### Fibrosis and inflammation-induced the upregulation of FGF10 expression in GO and non-GO OFs

**Fig 4:**
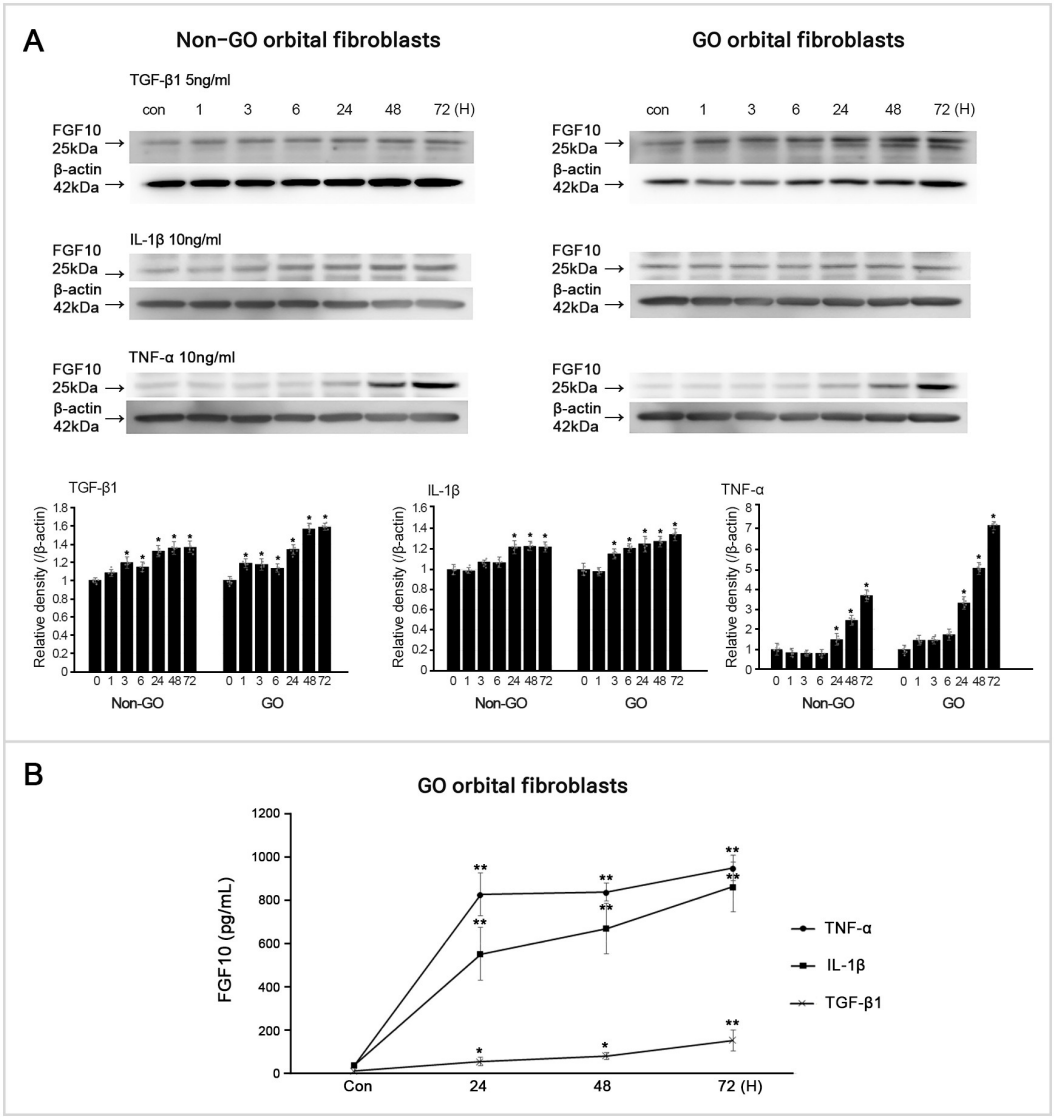
Effect of transforming growth factor (TGF)-β1, interleukin (IL)-1β, and tumor necrosis factor (TNF)-α on fibroblast growth factor 10 (FGF10) expression in Graves′ orbitopathy (GO) and non-GO orbital fibroblasts (OFs). (A) Representative western blot images showing that TGF-β1, IL-1β, and TNF-α increased expression of FGF10 at various time points (0–72 h) in both GO and non-GO OFs. Bar graphs show the relative density of each protein normalized to the level of β-actin and are represented as means ± standard deviation. (B) Expression levels in GO OFs of secreted FGF10 protein in response to TGF-β1, IL-1β, and TNF-α, using ELISA. Experiments were performed at least three times using different strains. *p < 0.05.

**Fig 5:**
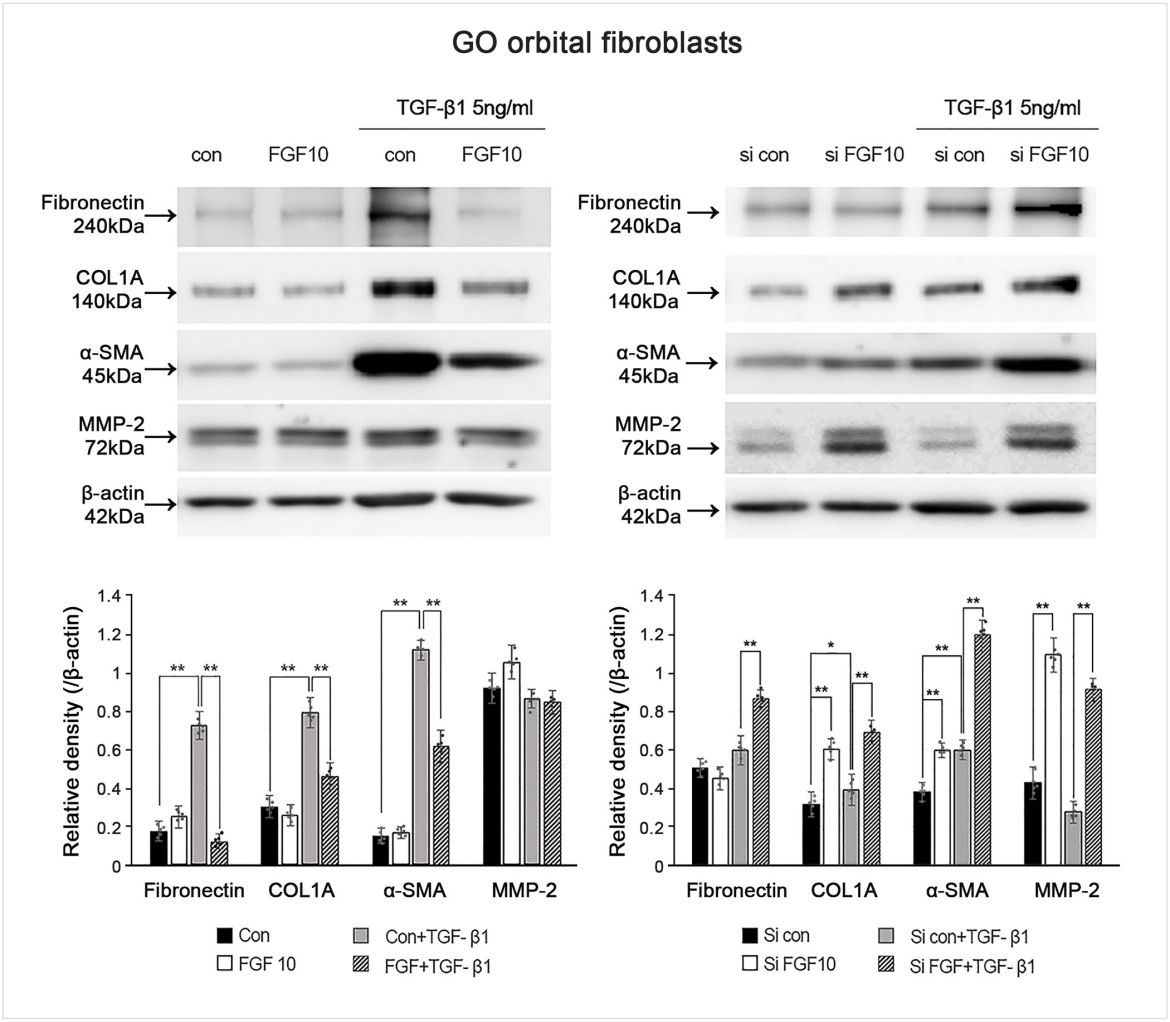
Effect of upregulation of fibroblast growth factor 10 (FGF10) using recombinant human (rh) FGF10 or blockage of FGF10 by small interfering (si) FGF10 transfection on transforming growth factor (TGF)-β1 induced fibrosis-related protein expression. The rhFGF10 suppressed the TGF-β1 induced fibronectin, collagen Iα (COL1A), and α-smooth muscle actin (α-SMA) expression in Graves′ orbitopathy (GO) orbital fibroblasts (OFs). Knockdown of FGF10 induced the increase of TGF-β1 induced fibronectin, COL1A, and α-SMA expression in GO OFs. Bar graphs show the relative density of each protein normalized to the level of β-actin and are represented as mean ± standard deviation. Experiments were performed in at least three cells cultured from different strains. *p < 0.05, **p < 0.01.

After treatment with 10 ng/mL of IL-1β, TNF-α, and 5 ng/mL of TGF-β on OFs, the protein levels were increased under inflammatory and fibrotic conditions (Fig 5). In addition, after treatment with IL-1β, TNF-α, and TGF-β for 72 h, we also found that the expression of FGF10 protein increased gradually in GO and non-GO OFs by western blot analysis in a time-dependent manner (Fig 4A).

Since FGF10 is a secreted molecule, we also determined secreted FGF10 expression levels in GO OFs under inflammatory and fibrotic conditions. After treatment with IL-1β, TNF-α, or TGF-β (24–72 h), FGF10 protein expression was significantly higher in GO OFs (Fig 4B).

### FGF10 overexpression or blockage altered TGF-β1 induced fibronectin, COLIA, α-SMA protein expression

To address the role of FGF10, we treated GO OFs with rhFGF10 or transfected with siFGF10 to change the expression levels of FGF10 in GO OFs. After treatment with rhFGF10 (100 ng/mL, 1 h) in GO OFs, TGF-β1 (5 ng/mL, 24 h) induced fibronectin, COL1A, and α-SMA protein expression substantially decreased (Fig 5). In contrast, knockdown of FGF10 via siFGF10 transfection (100 ng/mL, 24 h) induced an exaggerated increase in TGF-β-induced fibronectin, COL1A, α-SMA, and MMP-2 expression (Fig 5).

### Knockdown of FGF10 induced overexpression of IL-6, IL-8, and COX-2 proteins

**Fig 6:**
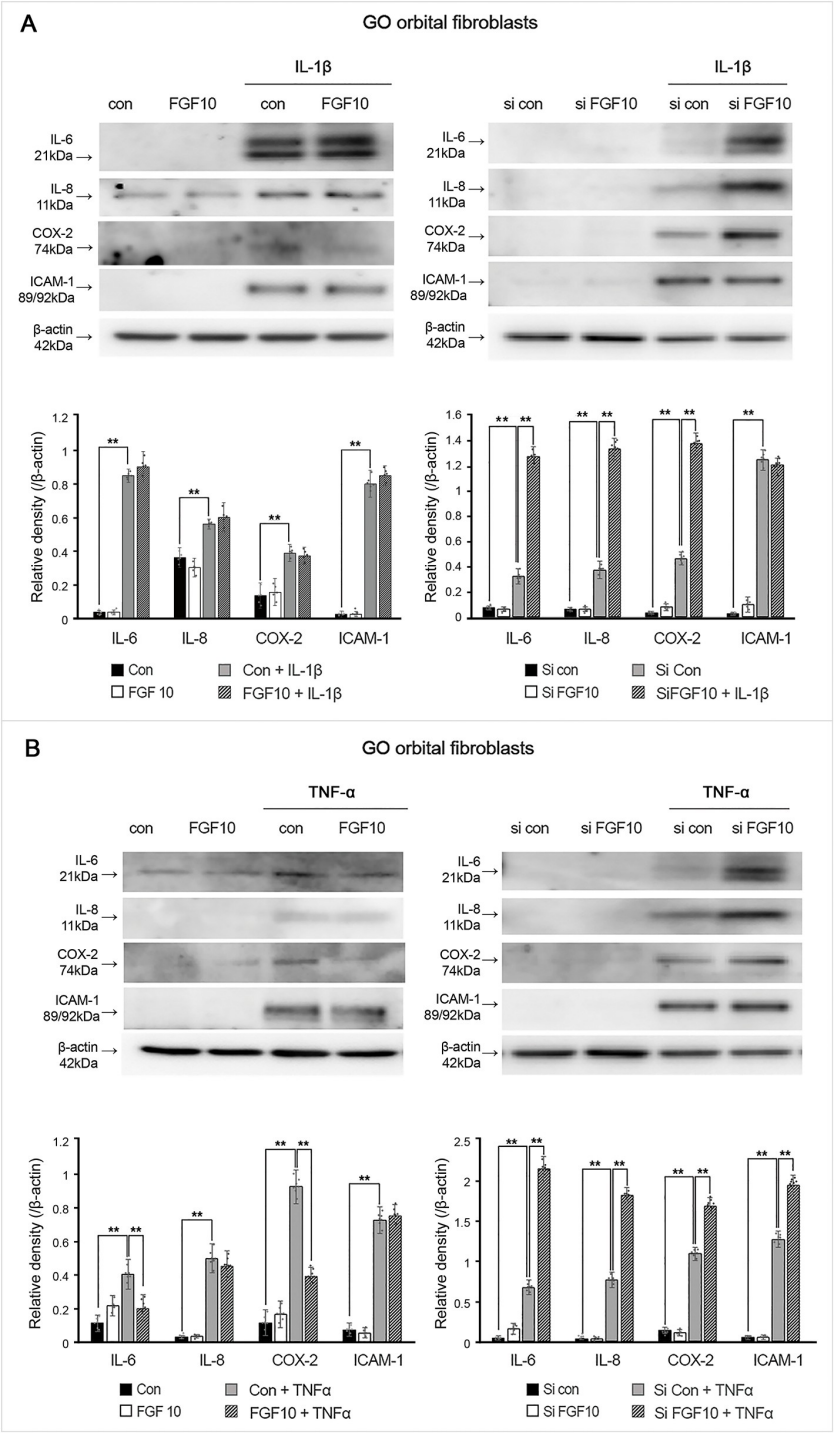
Effect of upregulation of fibroblast growth factor 10 (FGF10) using recombinant human (rh) FGF10 or blockage of FGF10 by small interfering (si) FGF10 transfection on interleukin (IL)-1β or tumor necrosis factor (TNF)-α induced proiflammatory protein expression. (A) FGF10 did not suppress IL-1β induced IL-6, IL-8, cyclooxygenase (COX)-2, and intercellular adhesion molecule-1 (ICAM-1) in Graves′ orbitopathy (GO) orbital fibroblasts (OFs). However, knockdown of FGF10 induced the increase of IL-1β induced IL-6, IL-8 and COX-2 expression in GO OFs. (B) FGF10 treatment attenuated expression of TNF-a induced IL-6 and COX-2 protein. However, knockdown of FGF10 induced the increase of TNF-α induced IL-6, IL-8, COX-2, and ICAM-1 expression. Bar graphs show the relative density of each protein normalized to the level of β-actin and are represented as mean ± standard deviation. Experiments were performed in at least three cells cultured from different strains. **p < 0.01.

We investigated whether FGF10 could contribute to the inflammatory response in GO OFs. IL-1β (10 ng/mL, 16 h) induced IL-6, IL-8, and COX-2 protein expression further increased in siFGF10 (100 ng/mL, 24 h) transfected OFs (Fig 6A). However, rhFGF10 (100 ng/mL) did not suppress IL-1β induced IL-6, IL-8, COX-2, and ICAM-1 protein production, probably because IL-1β was a too powerful inflammatory stimulant for FGF10 to compensate (Fig 6A).

In addition, we also treated GO OFs with TNF-α (10 ng/mL, 16 h) as another inflammatory stimulant (Fig 6B). Similar to IL-1β stimulated data, TNF-α upregulated the production of IL-6, IL-8, and COX-2 protein, which was further increased by blocking FGF10. Interestingly, the expression levels of IL-6 and COX-2 protein were attenuated by the treatment of FGF10.

### FGF10 upregulated phosphorylation of ERK in non-GO and GO OFs

**Fig 7:**
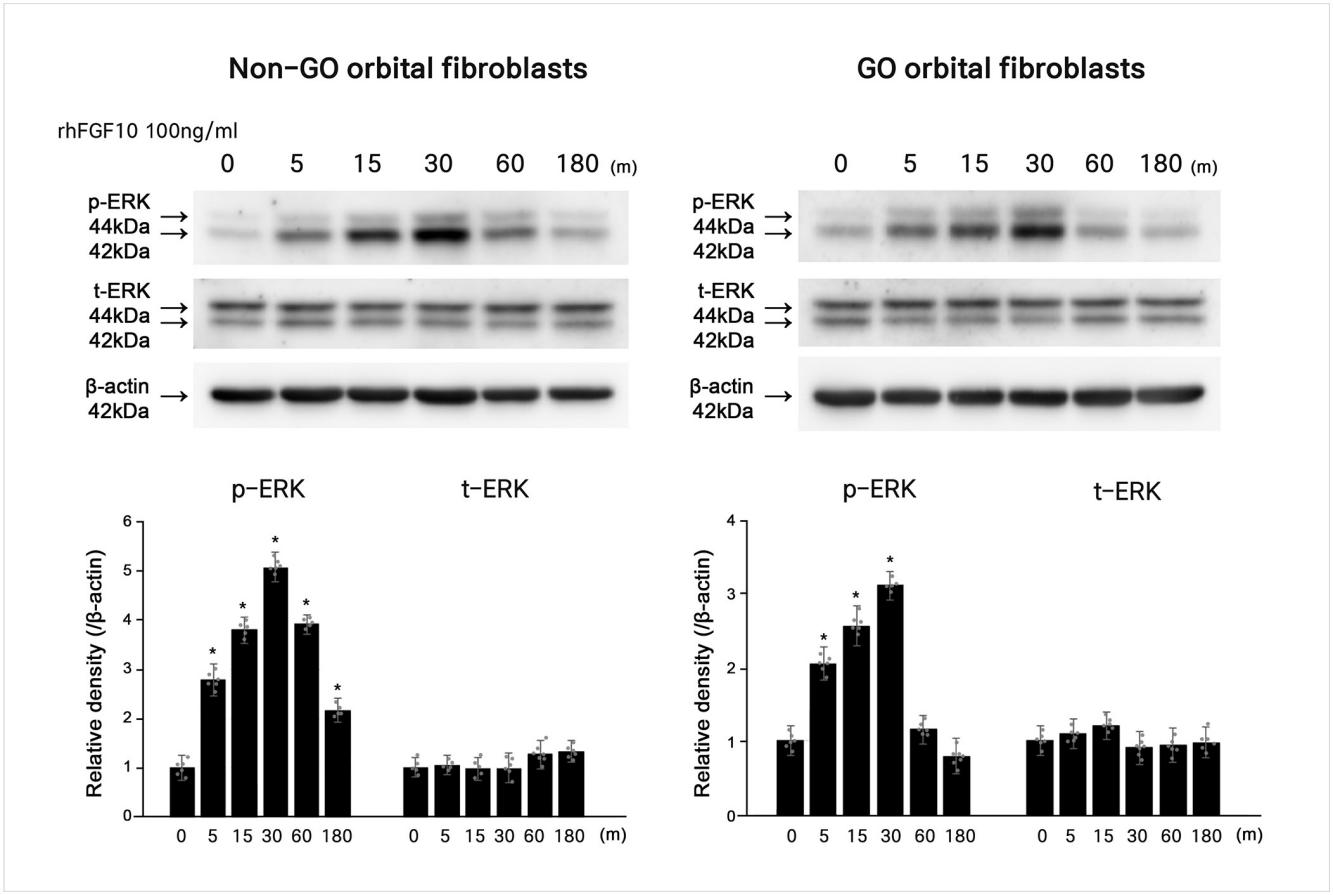
Role of fibroblast growth factor 10 (FGF10) in the phosphorylation of signaling molecule, extracellular signal-regulated kinase (ERK). The increase of phosphorylation of ERK was noted in rhFGF10 treated orbital fibroblasts from Graves′ orbitopathy (GO) and non-GO patients. The phosphorylation of ERK gradually increased and decreased after peaking at 30 minutes of treatment. Bar graphs show the relative density of each protein normalized to the level of β-actin and are represented as mean ± standard deviation. Experiments were performed in at least three cells cultured from different strains. *p < 0.05.

To identify signaling pathways affected by the regulation of FGF10, phosphorylation of ERK was assayed. rhFGF10 (100 ng/ml) treatment increased the levels of phosphorylated ERK signaling molecules in GO and non-GO OFs (Fig 7).

## Discussion

In this study, we investigated the roles of FGF10 in the pathogenesis of GO, especially in fibrosis and inflammation. First, we compared expression levels of FGF10 and FGFR2b between GO and non-GO tissues and cells; FGF10 was significantly downregulated in GO compared to non-GO specimens, whereas the mRNA transcript levels of *fibronectin*, *COL1A*, and *α-SMA* expression were higher in GO OFs than non-GO OFs. In addition, fibrotic and proinflammatory stimuli induced the upregulation of FGF10 expression in a time-dependent manner in both GO and non-GO OFs. TGF-β1 induced fibronectin, COL1A, and α-SMA protein expression in GO OFs was attenuated by rhFGF10 treatment; however, it was further increased by knockdown of FGF10 via siFGF10 transfection. Knockdown of FGF10 via siFGF10 increased the expression levels of IL-1β- and TNF-induced IL-6, IL-8, and COX-2 protein expression. Moreover, overexpression of FGF10 via rhFGF10 led to a decrease in the expression levels of TNF-α-induced IL-6 and COX-2. Based on these results, FGF10 might have a protective role not only in fibrosis and but also in inflammation as well. Considering the anti-inflammatory and anti-fibrotic role of FGF10, as shown in our data, the lack of FGF 10 expression in GO orbit may have induced or aggravated the GO phenotype.

FGF10 is a paracrine signaling growth factor of 215 amino acids and plays an important role during tissue homeostasis in adults [13]. Following injury, FGF10 robustly increases in the wound microenvironment and has further demonstrated some ability to promote wound healing *in vitro* and *in vivo*. Initial *in vitro* wound healing studies demonstrated that FGF10 promotes proliferation and differentiation of human keratinocytes, suggesting its likely role in epidermal growth and differentiation [21]. After analyzing the data regarding the role of FGF10 in fibrosis, it can be inferred that its role in the fibrotic process is complex because individual FGF family members may exert pro-and anti-fibrotic effects, depending on the responding cell type as well as the context of other signaling molecules [22]. Moreover, FGF subfamilies usually share FGFR. Among other FGFs that can share the receptor with the others, some members of FGF can act as ‘pro-fibrotic’ through their mitogenic activity on fibroblasts, while others have ‘anti-fibrotic’ effects by promoting epithelial cell regeneration [22]. Thus, the role of FGF10 in fibrosis is complex. For example, FGF10 levels were reduced in the lungs of aged mice before and after the injury and in idiopathic pulmonary fibrosis patients with the progressive disease compared with stable disease [23]. One study showed that FGF10 could prevent or reduce lung-specific inflammation/ fibrosis due to traumatic or infectious lung injury [24]. We, for the first time, demonstrated that FGF10 blocked TGF-β induced fibrosis-related protein expression in primary cultured OFs and also found diminished tissue level of FGF10 in GO tissues than in non-GO tissues. Recently, Sun et al. [15] reported that reduced expression of FGF10/FGFR2 was found in fibrotic retinal tissues and NRK52E cells with DBP exposure, and these findings are consistent with our results. However, there are contradictory results regarding FGF10 and fibrosis. In another study on FGFR antagonists, inhibition of endogenous FGFR2b ligands (FGF7 and FGF10) does not alter bleomycin-induced pulmonary fibrosis, suggesting that these ligands are not essential for fibrosis [25, 26].

Studies regarding the role of FGF10 in inflammation have shown that FGF10 is overexpressed in psoriatic skin, which is an autoimmune disease [27, 28]. FGF10 is known to protect neurons against oxygen-glucose deprivation injury in vitro, and FGF10 treatment depressed the triggered inflammatory factors of the TNF-α, IL-6, and nuclear factor-κB signaling pathways in a mouse middle cerebral artery occlusion model [29]. FGF10 treatment inhibited the release of pro-inflammatory cytokines after spinal cord injury in vivo [30]. Data regarding the effects of FGF10 on inflammation have also been contradictory. In one study, FGF10 promoted epithelial cell proliferation via stimulation of COX-2 expression and upregulation of prostaglandin E2 production [31]. In another, anti-FGF10 monoclonal antibody inhibited proliferation of human keratinocytes and reduced inflammation in propranolol-induced psoriasis-like lesions. Therefore, our findings may provide proof of principle that blockage of FGF10 can inhibit psoriasis-related inflammation [32].

In this study, although basal levels of FGF10 differed between GO and non-GO orbital tissues and primary cells, the inhibitory effect of FGF10 on fibrosis did not differ between GO and non-GO cells. According to previous studies [20, 33–35], GO and non-GO cells under primary culture conditions often show similar patterns of response to stimulation and inhibition of specific targets that may not reflect actual clinical events. Nonetheless, it is notable that our study found a significant difference in FGF10 expression levels between GO and non-GO tissues and OFs.

In conclusion, the precise mechanism by which FGF10 is involved in GO pathogenesis is not clear; however, the fibrotic and inflammatory processes were reduced by FGF10 treatment in the present study. Using a rabbit model of corneal CO_2_ laser injury, topical FGF-10 was shown to accelerate corneal epithelial wound healing and reduce inflammation, stromal edema, and fibrosis [32]. Although therapeutic approaches using exogenous FGFs, antibodies, or small molecules are still challenging, and many avenues of the investigation remain open, this study provides the concept that therapeutic strategies aimed directly at FGF10 could be beneficial in the treatment of GO.

## Supporting information

S1 Fig Full length gels representative of Western blot analysis for Figs 1 and 2. (TIF)

S2 Fig Full length gels representative of Western blot analysis for Fig 3. (TIF)

S3 Fig Full length gels representative of Western blot analysis for Figs 4 and 5. (TIF)

S4 Fig Full length gels representative of Western blot analysis for Figs 6 and 7. (TIF)
