# Supplementary figures and images for: Association of fibroblast growth factor 10 with the fibrotic and inflammatory pathogenesis of Graves’ orbitopathy
Source: PLoS One. 2021 Aug 12;16(8):e0255344. doi: 10.1371/journal.pone.0255344 (PMC8360584; doi:10.1371/journal.pone.0255344)

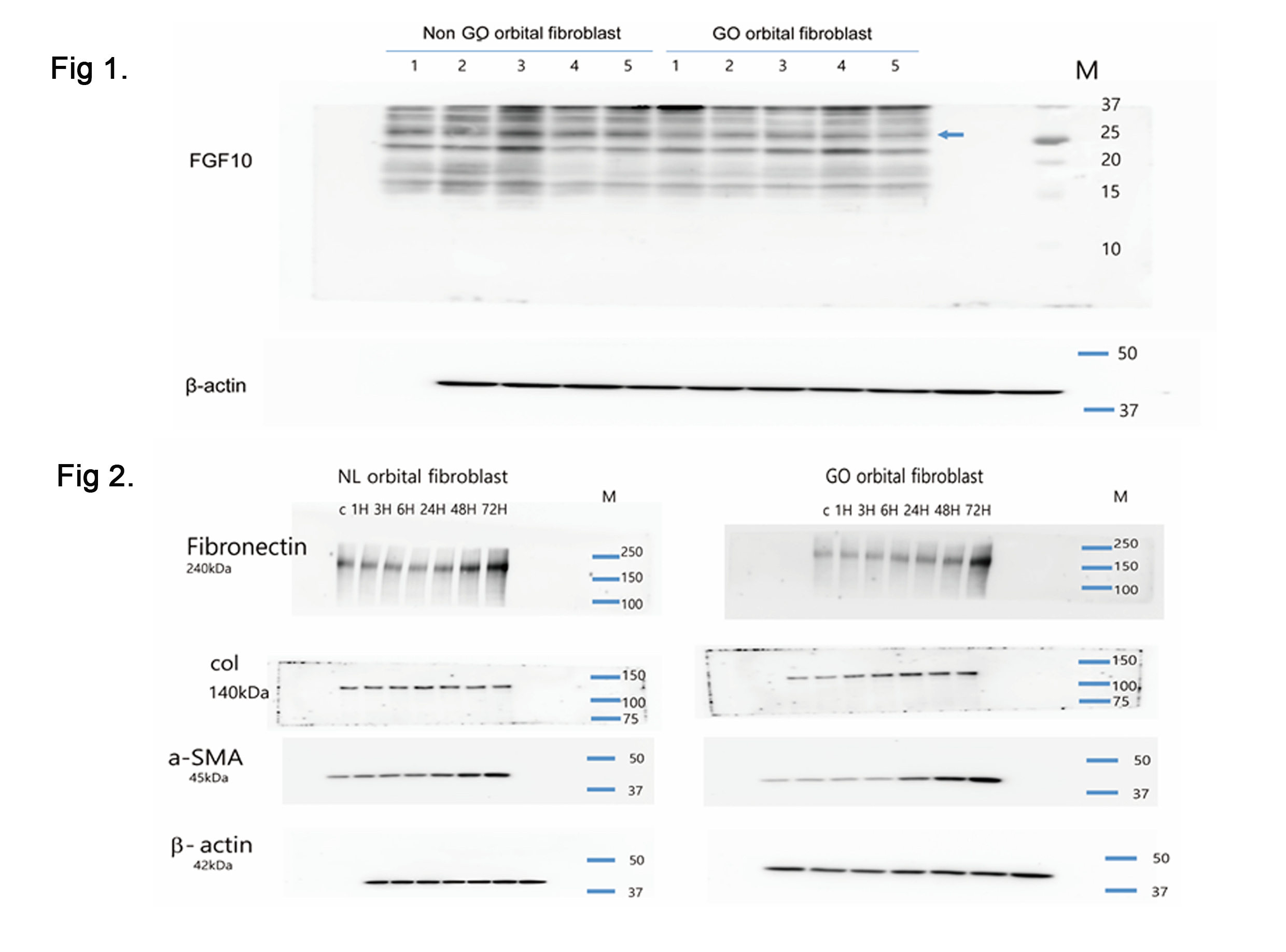

Supplement: S1 Fig — (TIF) [file pone.0255344.s001.tif]

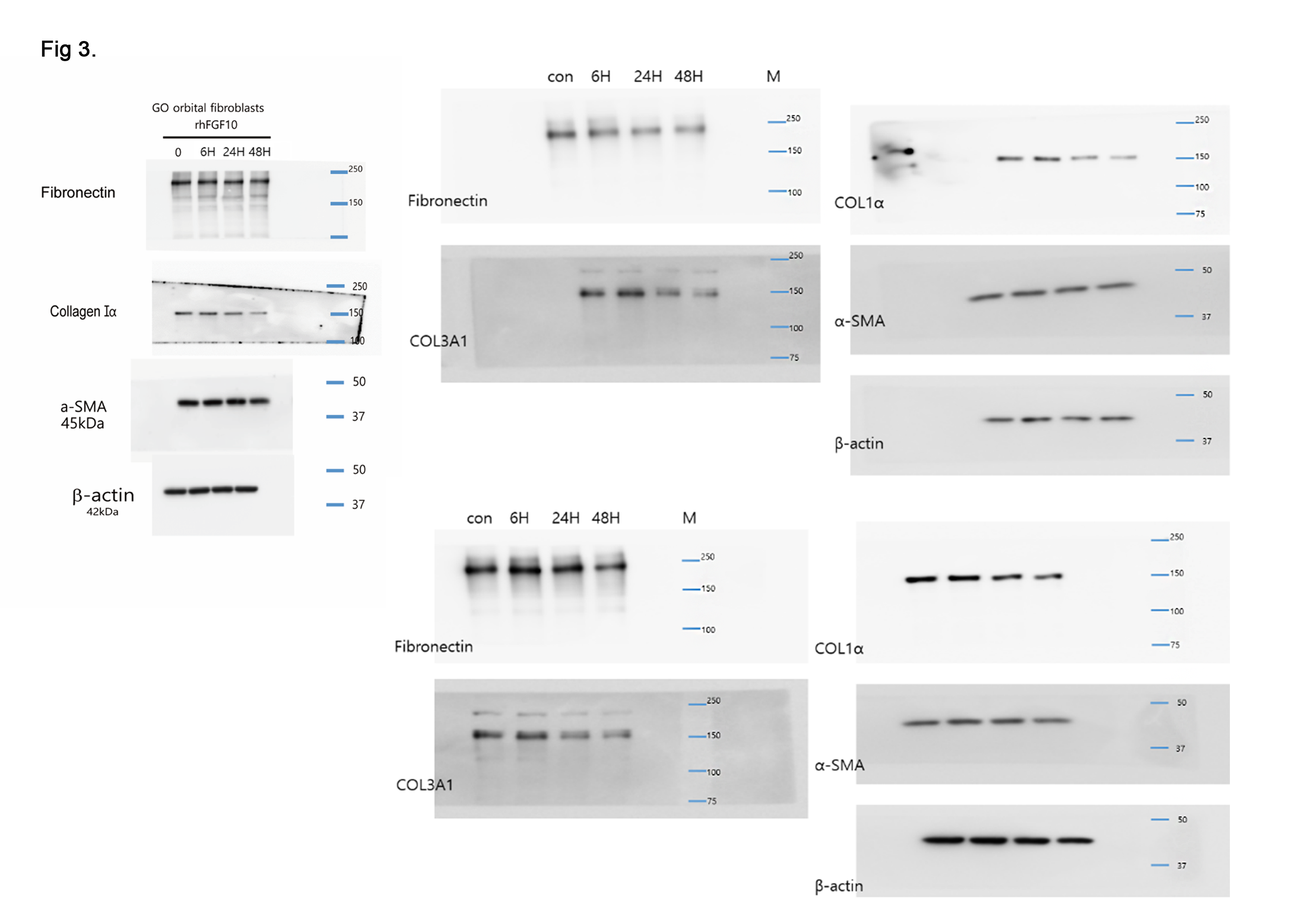

Supplement: S2 Fig — (TIF) [file pone.0255344.s002.tif]

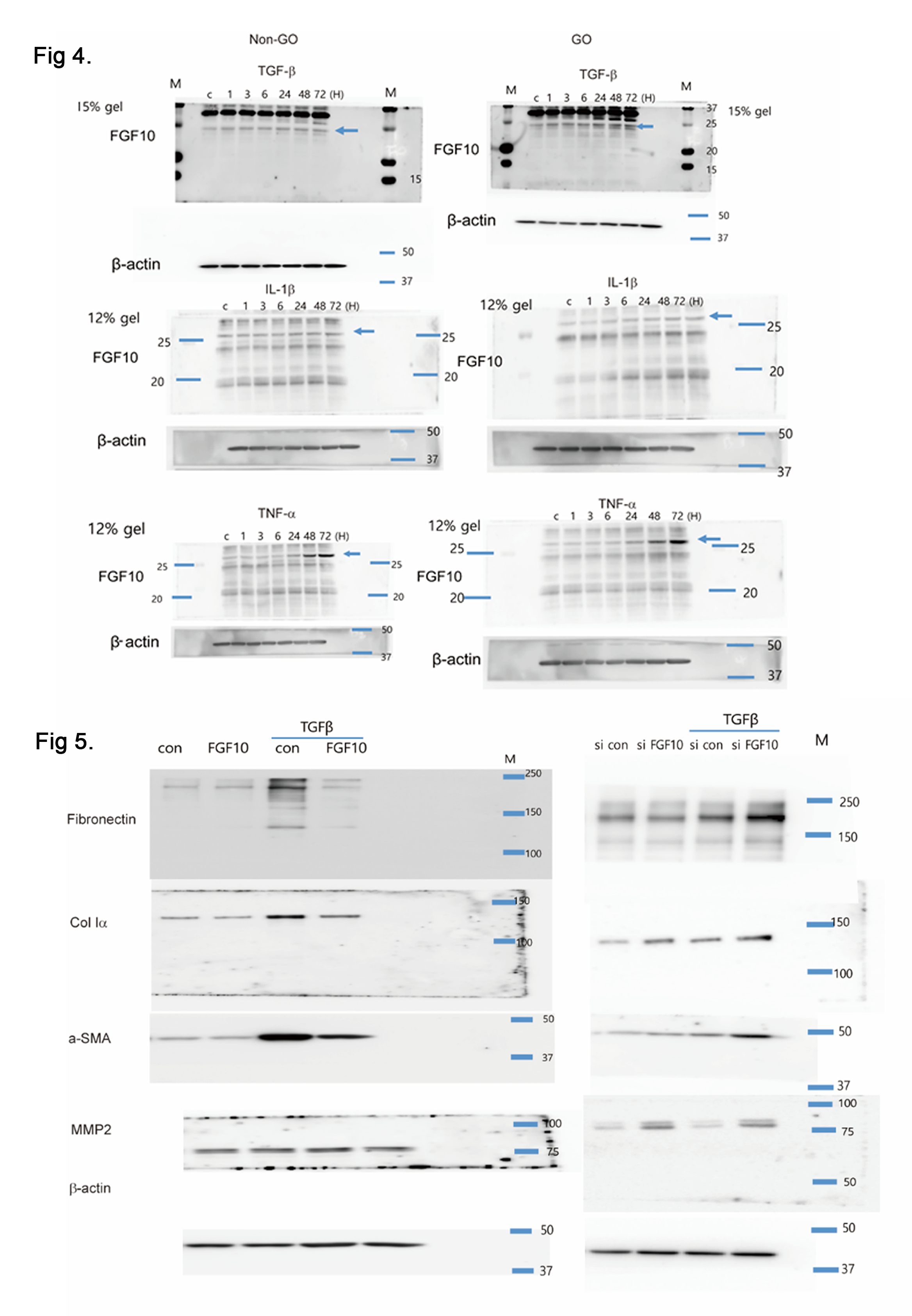

Supplement: S3 Fig — (TIF) [file pone.0255344.s003.tif]

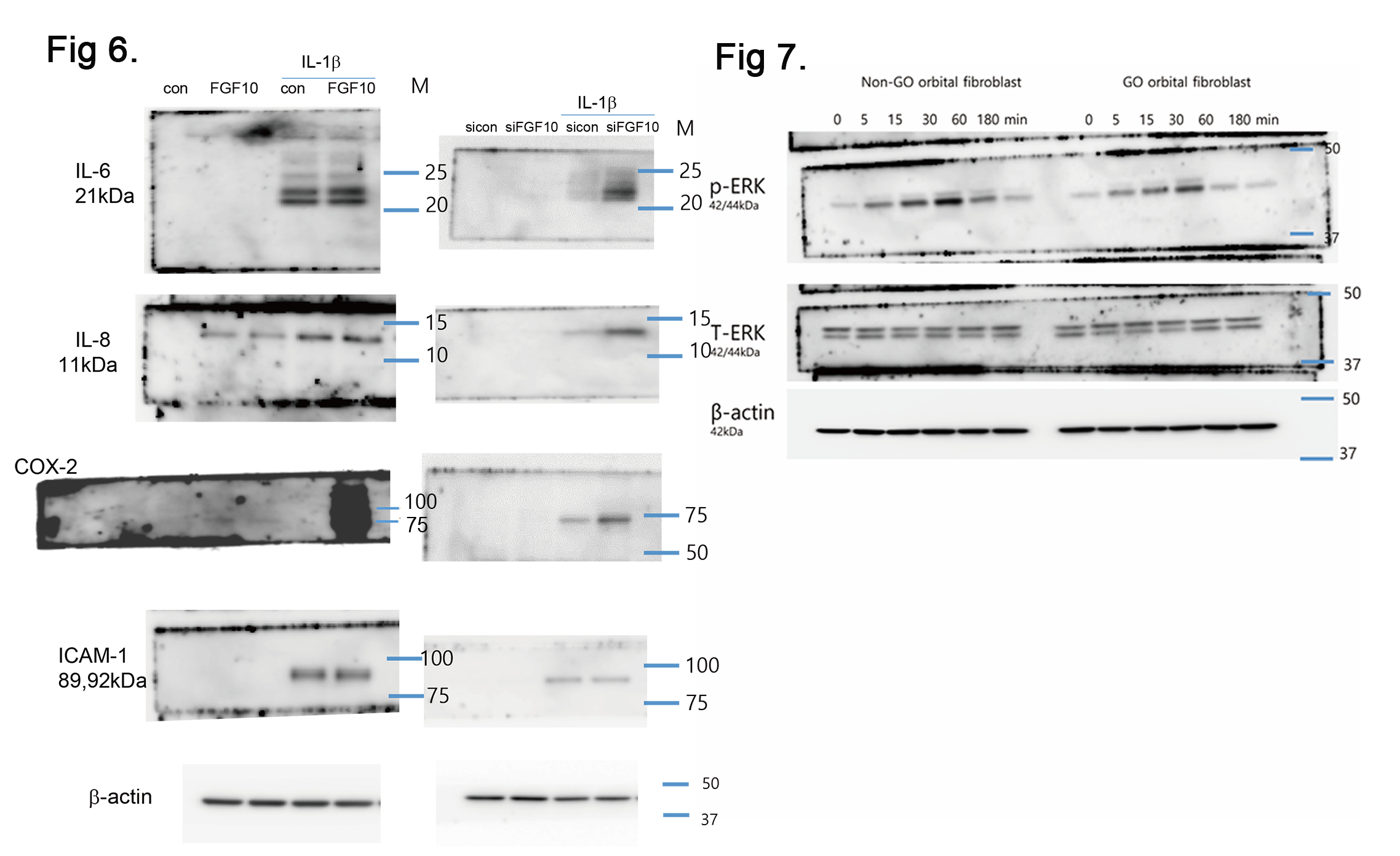

Supplement: S4 Fig — (TIF) [file pone.0255344.s004.tif]
